# Coated cysteamine and choline chloride could be potential feed additives to mitigate the harmful effects of fatty liver hemorrhagic syndrome in laying hens caused by high-energy low-protein diet

**DOI:** 10.1016/j.psj.2024.104296

**Published:** 2024-09-05

**Authors:** Muhammad Umar Yaqoob, Yingying Qi, Jia Hou, Li Zhe, Xiangde Zhu, Peng Wu, Zhefeng Li, Minqi Wang, Yan Li, Min Yue

**Affiliations:** ⁎ College of Animal Science, Zhejiang University, Hangzhou 310058, China; † Provincial Key Agricultural Enterprise Research Institute of King Techina, Hangzhou King Techina Feed Co., Ltd. Zhejiang Hangzhou 311107, China; ‡ Key Laboratory of Systems Health Science of Zhejiang Province, School of Life Science, Hangzhou Institute for Advanced Study, University of Chinese Academy of Sciences, Hangzhou, 310024, China

**Keywords:** Antioxidant potential, coated cysteamine, choline, fatty liver hemorrhagic syndrome, laying hen

## Abstract

The research aimed to examine the impact of coated cysteamine (**CS**) and choline chloride (**CC**) on relieving the pathological effects of fatty liver hemorrhagic syndrome (**FLHS**) in laying hens. FLHS was induced by a high-energy low-protein (**HELP**) diet. Ninety laying hens were equally divided into 5 treatments with 6 replicates per treatment (3 hens/replicate). The control treatment (**Cont**) was fed a basal diet, while the remaining treatments were fed a HELP diet. Under the HELP dietary plan, 4 treatments were set by a 2 × 2 factorial design. Two levels of CS (**CS−**: 0.00 mg/kg CS; **CS+**: 100 mg/kg diet) and 2 levels of choline (**CC−**: 1,182 mg/kg; **CC+**: 4,124 mg/kg) were set and named **CS−CC− (HELP), CS+CC−, CS−CC+** and **CS+CC+**. The liver of the CS**−**CC**−** (HELP) group became yellowish-brown and greasy, with hemorrhages and bleeding spots. Elevated (*P* < 0.05) plasma and hepatic ALT and AST and hepatic MDA levels, combined with reduced (*P* < 0.05) plasma and hepatic SOD and GSH-Px activities in the CS**−**CC**−** (HELP) group proved that FLHS was successfully induced. Dietary supplementation of CS, CC, or both (CS+CC+) in HELP diets relieved the pathological changes, significantly (*P* < 0.05) reduced the AST and ALT levels, and strengthened the antioxidant potential in laying hens under FLHS. The highest (*P* < 0.001) plasma adiponectin concentration was observed in the CS+CC**−** and lowest in the CS**−**CC**−** (HELP) group. In addition, CS and CC supplementation lowers the elevated levels of hepatic T-CHO and TG by increasing the HDL-C and reducing LDL-C levels (*P* < 0.05) than CS**−**CC**−** (HELP) group. CS supplementation, either alone or with CC, helps laying hens restore their egg production. It could be stated that CS and CC supplements could ameliorate the adverse effects of FLHS by regulating antioxidant enzymes activities, modulating the hepatic lipid metabolism, and restoring the production performance in laying hens. Hence, adding CS and CC could be an effective way to reduce FLHS in laying hens.

## INTRODUCTION

Fatty liver hemorrhagic syndrome (**FLHS**) is a widespread noninfectious, nutritional, and metabolic disorder affecting laying hens globally. It imposes a significant economic burden on the commercial layer industry by causing a sudden decline in egg production and increased mortality (Rozenboim et al., 2016; Tan et al., 2021) in apparently healthy laying hens. Although the exact mechanisms behind FLHS still require complete understanding, the clinical symptoms resemble those of non-alcoholic fatty liver disease (**NAFLD**), including oxidative stress, hepatic inflammation, apoptosis, autophagy (Shini et al., 2020; Wang et al., 2020), and insulin resistance (Zhang et al., 2008; Zhang et al., 2019). Therefore, enhancing oxidative potential and insulin sensitivity could potentially reduce the severity or occurrence of FLHS.

Cysteamine (**CS**), the aminothiol agent, has the potential commercial importance for livestock. It has been used as a growth stimulant in various livestock and avian species. It stimulates growth performance by influencing different factors in the somatotropic axis (Sagar et al., 1982). Also, it protects the cells from oxidative stress (Vasin, 2014) and could neutralize its effects in animals (Barnett and Hegarty, 2016). Enteric-coated CS has been reported to treat NAFLD by lowering the alanine aminotransferase (**ALT**), aspartate aminotransferase (**AST**) levels, and oxidative stress. It also involves lowering insulin resistance via increasing adiponectin in humans (Dohil et al., 2011,^2012^). Under oxidative stress, coenzyme-A degradation up-regulates CS biosynthesis to defend the cell from adverse effects (Kobayashi et al., 2006). Glutathione (**GSH**) serves as a cellular defense against oxidative damage, and a deficiency in this compound can potentially result in hepatocellular injury and fibrosis (Liu and Gaston Pravia, 2010). Under intracellular GSH depletion, CS promotes the intracellular transport of cysteine to boost GSH production (Besouw et al., 2013). The thiol group of CS also serves as an antioxidant (Haenen et al., 1989). Adiponectin is a kind of protein that is produced and secreted by adipocytes. It has anti-inflammatory, anti-diabetic, and anti-atherogenic properties that reduce insulin resistance (Achari and Jain, 2017). Adiponectin enhances liver fat oxidation and reduces fatty acid production (Xu et al., 2003). Fat accumulation in the hepatocytes and insulin resistance are highly involved in FLHS (Zhuang et al., 2019). Adiponectin can help to increase insulin sensitization by enhancing fatty acid oxidation, ultimately reducing triglyceride (**TG**) levels (Fruebis et al., 2001; Yamauchi et al., 2001). Noteworthy, CS could enhance plasma adiponectin concentration (Dohil et al., 2011). Based on the literature, CS seems to be a potential feed additive to alleviate the harmful effects of FLHS in laying hens.

Choline (trimethyl, β-hydroxy ethyl ammonium) performs three metabolic functions (Chan, 1984): a component of cell membranes, involved in fat metabolism, and a neurotransmitter precursor. Choline enhances the anti-oxidative potential through its metabolite, betaine, which is required to synthesize methionine (Zeisel and Blusztajn, 1994). Additionally, choline is the component of phosphatidylcholine (Chan, 1984) that is needed for very low-density lipoprotein synthesis (Aziza et al., 2019), which transfers fat from the liver to blood (Vance, 2008). Choline has been reported to alleviate fatty liver in laying hens (Wolford and Polin, 1975).

However, the effects of dietary microencapsulated CS and/or extra addition of choline based on the recommended dosage workable for FLHS in laying hens are unknown. Hence, it was hypothesized that these active feed additives could offer the potential to alleviate FLHS caused by the high-energy low-protein (**HELP**) diet through the reduction of lipid accumulation and oxidative stress.

## MATERIALS AND METHODS

The experiment was approved by the Zhejiang University and Hangzhou King Techina Feed Co., Hangzhou, for ethical use and care of experimental hens.

### Experimental Birds and Treatments

The present experiment was conducted on 90 laying hens (breed: Hy-line Brown; age: 18 wk; average BW: 1.63 kg), acclimatized for 2 wk. Basal diet and fresh water were available freely to all hens during the adaptation period. Hens were assigned to 5 treatments, with 6 replicates per treatment, stratified based on their initial body weight. Each replicate consisted of 3 hens placed in a ladder-type cage made of stainless steel. The control (**Cont**) group was provided a basal diet (crude protein, CP: 15.86%; metabolic energy, ME: 2,678.99 kcal/kg) formulated as per recommendations of the National Research Council (1994). The remaining hens were divided into 4 treatments under 2 × 2 factorial design, where 2 levels of cysteamine (CS-Plus, 27% microencapsulated cysteamine HCl, King Techina, Hangzhou, China): 0 mg/kg (**CS−)** or 100 mg/kg (**CS+)** and 2 levels of choline (60% choline chloride): 1,182 mg/kg (**CC−**; as per recommendations of Hy-Line Brown 2018) or 4,124 mg/kg (**CC+**) were used. The 4 treatments were named; **CS−CC−** (CS: 0.00 mg/kg; CC 1,182 mg/kg); **CS+CC−** (CS: 100 mg/kg; CC 1,182 mg/kg); **CS−CC+** (CS: 0.00 mg/kg; CC 4,124 mg/kg); **CS+CC+** (CS: 100 mg/kg; CC 4,124 mg/kg) and fed on a high-energy low-protein diet (HELP; CP 12.00 %: ME 3,100.00 kcal/kg) to induce the FLHS as reported previously (You et al., 2023; Guo et al., 2024). Among those 4 groups, CS−CC− was a typical HELP diet model. The compositions of diets are presented in Supplementary Table 1.

### Data Recording

All laying hens were weighed on arrival at the experimental site to maintain the average body weight of hens in each replicate. Data recording about weekly feed intake and daily egg production was started after the adaptation period. The number of unqualified eggs (broken, malformed, soft-shelled, and dirty eggs) and mortality, if any, were recorded daily for each replicate. Data on feed intake was presented as average daily feed intake (**ADFI**), while data on egg production was used to calculate hen-day egg production (**HDEP**), hen-housed egg production (**HHEP**), number of hen-housed eggs (**HHE**), egg mass per bird, and egg mass per day. The data of total feed intake (kg) and total egg weight (kg) produced by a hen were used to calculate the feed:egg ratio. Data on production performance was divided into two phases: 1–6 and 7–13 wk of the experiment.

### Egg Quality Analysis

On d 30th, 60th, and 90th of the experiment, one egg per replicate was collected and analyzed for different egg quality indexes (albumen height, haugh unit, yolk color, and eggshell strength). DET-6000 from Nabel Co., Ltd., Kyoto, Japan, was used for egg quality analysis. Yolk weight was recorded separately, and the yolk:egg ratio was calculated. A digital vernier caliper (Guanglu Instruments Co., Ltd., Guilin 541004, P.R.China) was used to measure the eggshell thickness.

### Slaughtering and Sampling

All hens were off-feed for 12 h before slaughtering, and freshwater was provided ad libitum. Six laying hens (1 hen/ replicate) with average body weight were selected and marked for each treatment. Blood specimens were obtained from the jugular vein in heparinized tubes aseptically before slaughtering. Tubes containing blood were centrifuged at 3,500 rpm for 10 min to separate the plasma using a centrifuge machine (TD4-Bioridge, Shanghai Lu Xiangyi centrifuge instrument Co., Ltd., Shanghai, China). Samples of plasma were preserved at −20°C till further analysis. Marked laying hens were slaughtered by cutting the jugular vein after recording the live weight. Gross liver was separated, weighed, and imaged. Liver tissue samples (approximately 1 cm each) were taken and fixed in 4% paraformaldehyde for histopathological evaluation. Abdominal fat weight was recorded. Liver tissue was also stored in small tubes, put in dry ice, immediately shifted to the lab, and kept in a −20°C freezer till further analysis (Zhou et al., 2023).

### Antioxidant and Biochemical Analyses

Liver samples were used to prepare liver tissue homogenate. Plasma and liver homogenate samples were analyzed to assess the antioxidant status by determining the total antioxidant capacity (**T-AOC**) and enzymatic activities of catalase (**CAT**), total superoxide dismutase (**SOD**), malondialdehyde (**MDA**), and glutathione peroxidase (**GSH-Px**). Additionally, the liver homogenate and plasma samples underwent examination for triglyceride (**TG**), total cholesterol (**T-CHO**), low-density lipoprotein cholesterol (**LDL-C**), and high-density lipoprotein cholesterol (**HDL-C**) levels. In addition, plasma and hepatic concentrations of ALT and AST were analyzed. All antioxidant and biochemical indexes were analyzed using commercially available kits according to their operating instructions (Nanjing Jiancheng Biological Engineering Institute, China). A chicken adiponectin ELISA kit (Jiangsu Meimian Industrial Co. Ltd., Jiangsu, China) was used to determine the plasma adiponectin concentration following the kit instructions.

### Pathological Analysis

Liver samples for analysis were fixed in 4% paraformaldehyde. Subsequently, hepatic histopathology was assessed using Hematoxylin-eosin (**H&E**) staining. Different pathological conditions were represented with arrows on H&E slides: red arrows: inflammatory lymphocytes; yellow arrows: steatosis of liver cells; blue arrows: liver cell edema; black arrows: focal necrosis of liver cells. In addition, based on the 4-point scoring system (Mann et al., 2012), each slide was given a score point based on hepatocellular degeneration, inflammatory cells, and necrosis. Details of the 4-point scoring system have been provided in Supplementary Table 2. Oil Red O staining (Yamamoto et al., 2018) was performed to analyze the hepatic fat accumulation. Images were analyzed through a digital pathological image analysis software (Aipathwell) based on artificial intelligence learning form Servicebio.

### Statistical Analysis

The data were analyzed through SPSS 16.0, in which dietary treatments served as independent variables and one dietary treatment as the experimental unit, while each replicate served as the statistical unit. The data collected from treatments with HELP diets were subjected to a T-test in comparison to the control group. Simultaneously, a thorough 2-factor analysis was conducted to analyze the effects of main factors (CS or CC) and their interaction. Tukeys’ test was conducted to analyze the significant difference between the 4 HELP treatments when there existed significant interaction difference between the main factors, where *P* < 0.05 was set as a significant difference, and *P* < 0.10 showed a tendency.

## RESULTS

### High-Energy Low-Protein diet and FLHS Model

**Figure 1:**
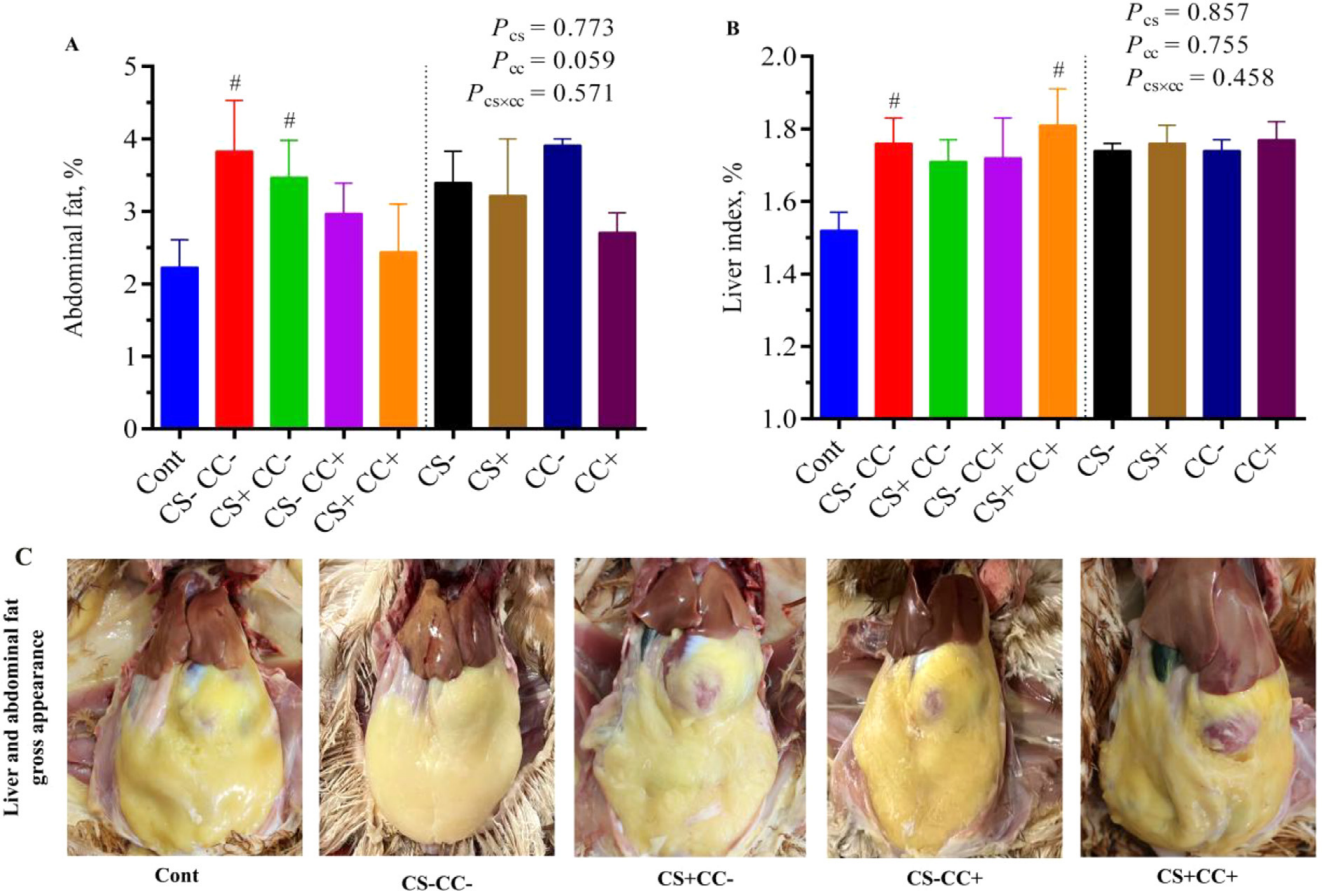
Effect of coated cysteamine with or without choline chloride on abdominal fat percentage (**A**) and liver index percentage (**B**) in laying hens under FLHS induced by HELP diet. The lower side of the picture represents the liver and abdominal fat gross appearance of the representative hen of each group (**C**). In bar graphs data are presented as mean ± SEM, n = 6/group. The left side of each graph shows a single-factor analysis, while the right side shows a 2-factor analysis. ^#^*P* < 0.05 shows the difference from the control group based on T-test. CS**−** and CS+ without and with (100 mg/kg) cysteamine supplementation. CC**−** (choline 1,182 mg/kg) and CC+ (choline 4,124 mg/kg) without and with supplementation of choline chloride, respectively. CS**−**CC**−** was a typical high-energy low-protein diet model.

**Figure 2:**
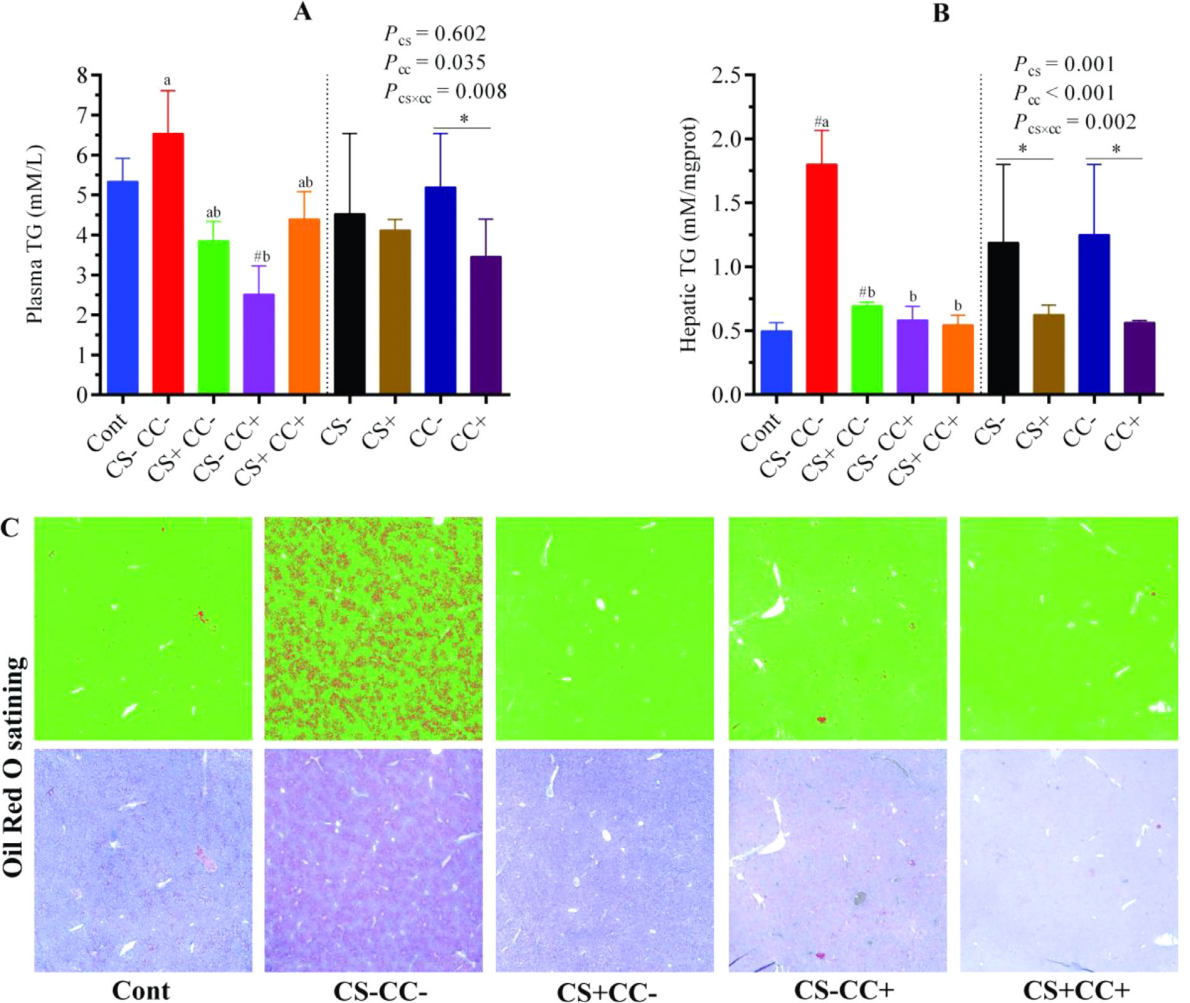
Effect of coated cysteamine with or without choline chloride on lipid accumulation in plasma and liver. (**A** and **B)** represent the plasma and hepatic triglyceride concentrations, respectively. In addition, Oil Red O staining was performed to visualize the fat accumulation in the live tissues (**C**). The red area under each slide is proportional to the concentration of fat accumulation in liver tissue. In bar graphs data are presented as mean ± SEM, n = 6/group. The left side of each graph (**A** and **B**) shows a single-factor analysis, while the right side shows a 2-factor analysis. On the left side, ^#^*P* < 0.05; shows the difference from the control group according to the T-test. At the same time, different superscripts (a and b) indicate significant differences among the 4 HELP diet groups when there was significant interaction between CS and CC. According to 2 factors analysis from CS and CC, an asterisk (*) shows differences between the groups. CS**−** and CS+ without and with (100 mg/kg) cysteamine supplementation. CC**−** (choline 1,182 mg/kg) and CC+ (choline 4,124 mg/kg) without and with supplementation of choline chloride, respectively. CS**−**CC**−** was a typical high-energy low-protein diet model.

**Figure 3:**
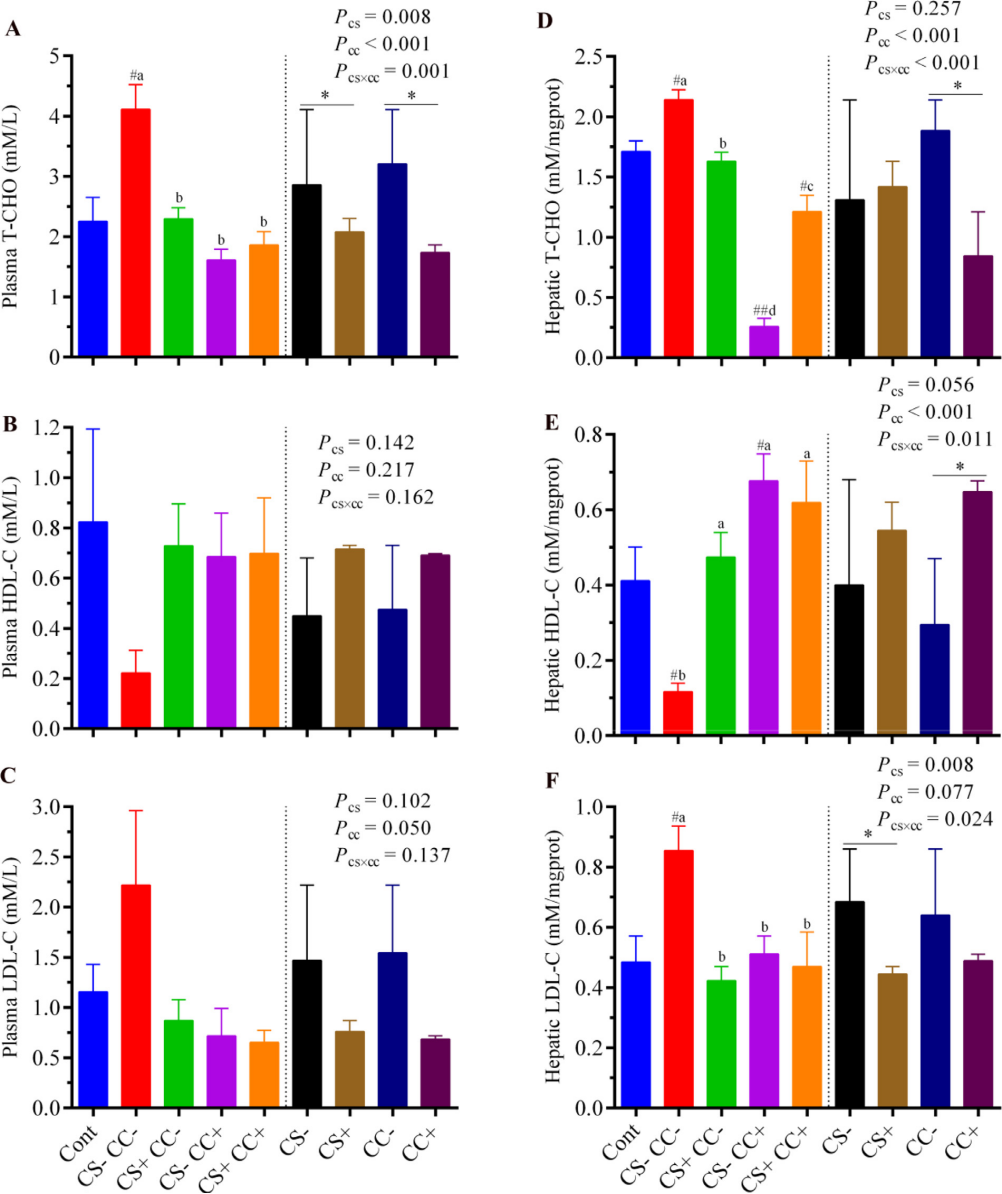
Effect of coated cysteamine with or without choline chloride on indexes relative to total cholesterol and lipid metabolism-protein concentrations in plasma and liver. (**A)** plasma T-CHO; (**B)** plasma HDL-Cl; (**C)** plasma LDL-C; (**D)** hepatic T-CHO; (**E)** hepatic HDL-C; (**F)** hepatic LDL-C. Data are presented as mean ± SEM, n = 6/group. The left side of each graph shows a single-factor analysis, while the right side shows a 2-factor analysis. On the left side, ^#^*P* < 0.05; ^##^*P* < 0.001 shows the difference from the control group according to the T-test. At the same time, different superscripts (a, b, and c) indicate significant differences among the 4 HELP diet groups when there was significant interaction between CS and CC. According to 2 factors analysis from CS and CC, an asterisk (*) shows differences between the groups. CS**−** and CS+ without and with (100 mg/kg) cysteamine supplementation. CC**−** (choline 1,182 mg/kg) and CC+ (choline 4,124 mg/kg) without and with supplementation of choline chloride, respectively. CS**−**CC**−** was a typical high-energy low-protein diet model.

The CS-CC- (HELP) group exhibited notably higher (*P* < 0.05) percentages of abdominal fat and liver index (Figure 1), hepatic TG deposition (Figure 2B), T-CHO in plasma, T-CHO and LDL-C levels in the liver (Figure 3A, 3D, and 3F), elevated ALT and AST levels in plasma and liver homogenate (Figure 5 B–5E), and decreased hepatic HDL-C level (Figure 3E), accompanied by reduced antioxidant capacity (Table 1) as compared to the Cont group. Additionally, hens in the CS-CC- (HELP) group displayed a liver with a greasy and yellowish-brown appearance, along with evident hemorrhages and bleeding spots. H&E staining revealed inflammatory lymphocytes in the portal area, multi-focal necrosis of liver cells in the parenchyma and surrounding blood vessels, nuclear pyknosis, lymphocyte infiltration, hepatic steatosis, and blood vessel congestion in the liver tissue of the CS-CC- (HELP) group. These findings collectively indicate that the HELP diet effectively induced FLHS.

**Table 1:** Effect of coated cysteamine with or without choline chloride on antioxidant capacity in laying hens under FLHS induced by HELP diet.

		Plasma (corresponding units/mL)					Liver homogenate (corresponding units/mg prot)				
Treatments		T-AOC, mM	MDA, nmol	CAT, U	SOD, U	GSH-Px, U	T-AOC, mM	MDA, nmol	CAT, U	SOD, U	GSH-Px, U
Control		0.379	4.905	4.091	111.28	3819.50	4.347	5.890	26.195	138.803	32.601
CC**−**	CS**−** (HELP)	0.324	5.238	2.978	104.85*	3235.02*^b^	2.171**	10.959*^a^	18.832*^b^	87.960*	22.427*^b^
	CS+	0.311	4.952	3.711	107.24*	4190.36^a^	3.085*	7.036^bc^	31.681*^a^	110.567	33.750^a^
CC+	CS**−**	0.365	5.000	2.951	109.25	3983.79^a^	3.097*	6.320^c^	28.370^a^	89.916*	30.342^a^
	CS+	0.419	4.000	4.353	108.08	3966.97^a^	3.014*	9.969*^ab^	31.269**^a^	88.117*	29.931^a^
SEM		0.019	0.213	0.285	1.067	162.167	0.348	1.021	2.335	9.895	1.976
Means of main effects	CS**−**	0.344	5.119	2.964	107.05	3609.41^x^	2.634	8.640	23.601^x^	88.938	26.385^x^
	CS+	0.365	4.476	4.032	107.66	4078.66^y^	3.050	8.502	31.475^y^	99.342	31.841^y^
	CC**−**	0.318	5.095	3.345	106.05^n^	3712.69	2.628	8.998	25.257^m^	99.263	28.088
	CC+	0.392	4.500	3.652	108.66^m^	3975.38	3.056	8.144	29.820^n^	89.016	30.137
*P*-value											
Main factors	CS	0.261	0.087	0.146	0.613	0.001	0.133	0.864	<0.001	0.190	0.004
	CC	0.641	0.111	0.670	0.042	0.051	0.122	0.290	0.002	0.197	0.239
	CS×CC	0.815	0.329	0.642	0.153	0.001	0.075	<0.001	0.001	0.128	0.002

Each group had 6 replicates (n = 6/group). *P < 0.05; **P < 0.001 shows the difference from the control group based on T-test. “x and y” and “n and m” show the difference within factor CS and CC, respectively. Whereas a, b, and c were used to show the difference among the 4 HELP groups when there was significant interaction CS×CC. CS**−** and CS+ without and with (100 mg/kg) cysteamine supplementation. CC**−** (choline 1,182 mg/kg) and CC+ (choline 4,124 mg/kg) without and with supplementation of choline chloride, respectively.

### Liver Index, Fat Deposition and Metabolism

Figure 1A demonstrates that the CS+CC− (*P* < 0.05) group exhibited higher abdominal fat percentage compared to the Cont group. However, no significant (*P* > 0.05) difference was found in CS−CC+ and CS+CC+ groups for abdominal fat percentage compare to the Cont group. Similarly no difference (*P* > 0.05) was observed for CS+CC− and CS−CC+ groups for liver index percentage than the Cont group (Figure 1B). In comparison to the Cont, the CS−CC− group demonstrated a 71.24% increase in abdominal fat percentage (*P* < 0.05; Figure 1A) and a 16.25% increase in liver index (*P* < 0.05; Figure 1B). Analysis of the main factors indicated that the addition of CC tended (*P* = 0.059) to decrease abdominal fat percentage. However, there were no significant differences in other factors and their interactions for abdominal fat and liver index percentages (*P* > 0.05) were observed.

The analysis of the main factors exhibited that supplementation of CS significantly (*P* < 0.05) decreased the hepatic TG contents. In contrast, supplementation of CC significantly (*P* < 0.05) decreased the plasma and hepatic TG levels (Figures 2A and 2B). A significant (*P* < 0.05) interactive effect of CS×CC showed that hepatic TG level was decreased in the CS+CC−, CS−CC+, and CS+CC+ groups as compared with the CS−CC− (HELP) group (Figure 2B); however, plasma TG was only lower in the CS−CC+ than in the CS−CC− (HELP) group (Figure 2A). In addition, the Oil Red O staining showed that the fat droplet area ratio was decreased in the liver tissue of CS**−**CC+ and CS+CC+ groups (Figure 2C).

The analysis of the main factors exhibited that supplementation of CS significantly (*P* < 0.05) decreased the plasma T-CHO contents (Figure 3A). Meanwhile, supplementation of CC significantly (*P* < 0.05) decreased the plasma and hepatic T-CHO levels (Figures 3A–3D). A significant (*P* < 0.05) interactive effect of CS×CC showed that both plasma and hepatic T-CHO levels were decreased in the CS+CC−, CS−CC+, and CS+CC+ groups as compared with the CS−CC− (HELP) group. The analysis of the main factors for lipid metabolites showed that supplementation of CC significantly (*P* < 0.05) increased hepatic HDL-C, while supplementation of CS significantly (*P* < 0.05) decreased the hepatic LDL-C (Figures 3E–3F). A significant (*P* < 0.05) interactive effect of CS×CC showed that hepatic LDL-C levels were reduced while HDL-C was increased in the CS+CC−, CS−CC+, and CS+CC+ groups as compared with the CS−CC− (HELP) group (Figures 3E–3F).

**Figure 4:**
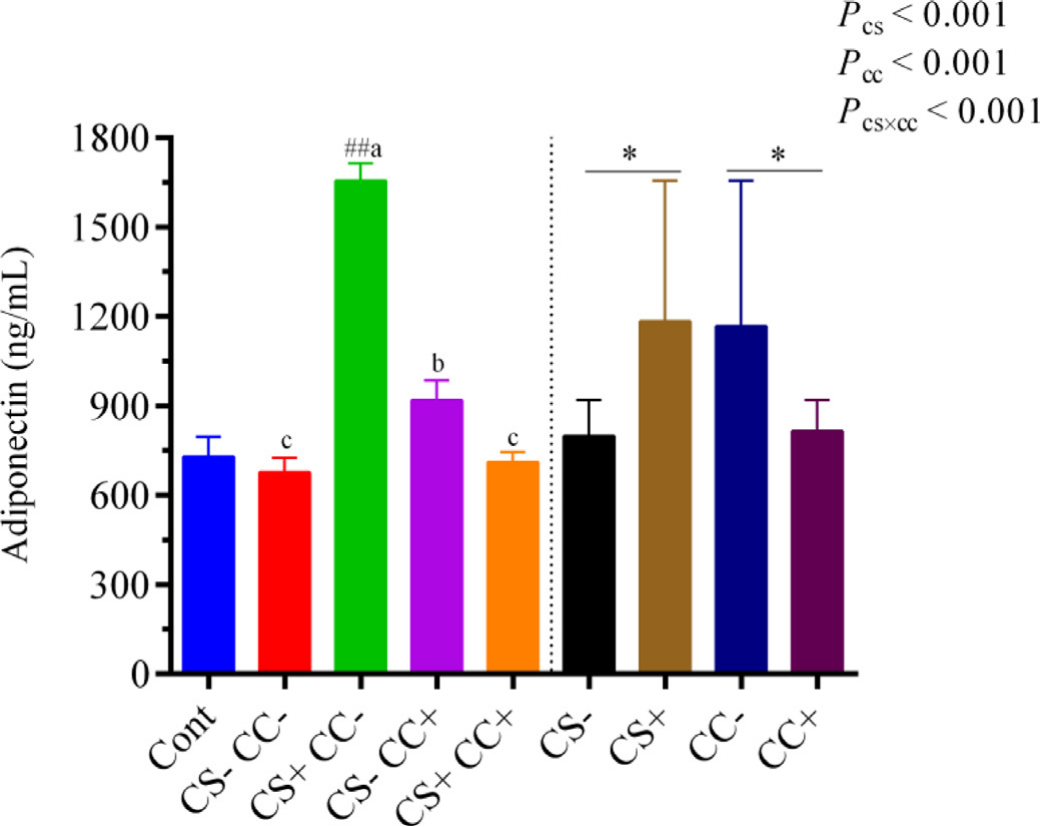
Plasma adiponectin concentration. Data are presented as mean ± SEM, n = 6/group. The left side of the graph shows a single-factor analysis, while the right side shows a 2-factor analysis. On the left side, ^##^*P* < 0.001 shows the difference from the control group according to the T-test. At the same time, different superscripts (a, b, and c) indicate significant differences among the 4 HELP diet groups when there was significant interaction between CS and CC. According to 2 factors analysis from CS and CC, an asterisk (*) shows differences between the groups. CS**−** and CS+ without and with (100 mg/kg) cysteamine supplementation. CC**−** (choline 1,182 mg/kg) and CC+ (choline 4,124 mg/kg) without and with supplementation of choline chloride, respectively. CS**−**CC**−** was a typical high-energy low-protein diet model.

Figure 4 presented a numerical (*P* > 0.05) reduction in the plasma adiponectin concentration of the CS−CC− (HELP) than the Cont group. While its concentration was significantly (*P* < 0.05) increased in the CS+CC− group than in the Cont group. The analysis of the main factors showed that plasma adiponectin level was significantly (*P* < 0.05) enhanced and reduced with supplementation of CS and CC, respectively. A significant (*P* < 0.05) interactive effect of CS × CC showed that adiponectin level was enhanced in the CS+CC− and CS−CC+ groups than the CS−CC− (HELP) group (Figure 4).

### Hepatic Health Status

**Figure 5:**
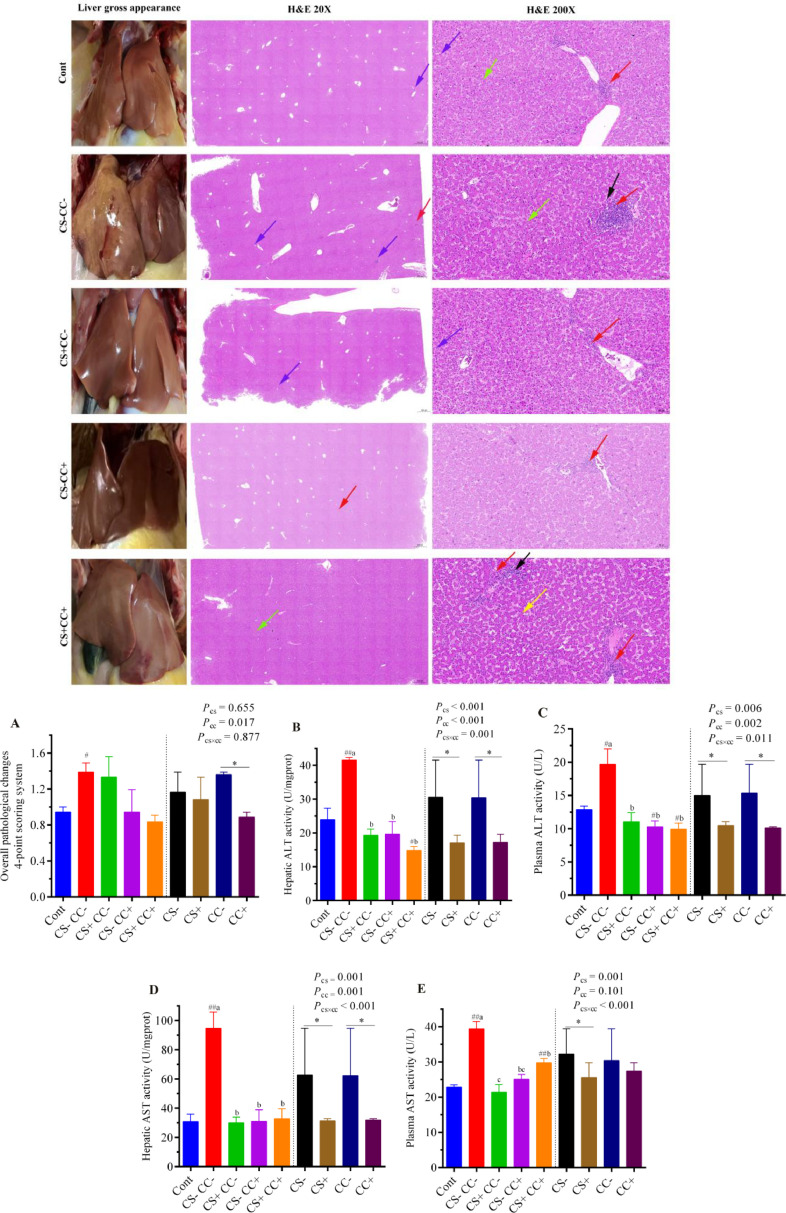
Effect of coated cysteamine with or without choline chloride on hepatic health. The figure represents the liver gross appearance of the representative hen of each group. H&E staining (20X and 200X) was performed to evaluate the hepatic histopathology. Arrows on H&E slides represent different pathological conditions: red arrows: inflammatory lymphocytes; yellow arrows: steatosis of liver cells; blue arrows: liver cell edema; black arrows: focal necrosis of liver cells. (**A)** represents the overall hepatic health status (the 4-point scoring system) based on hepatocellular degeneration, inflammatory cells, and necrosis. (**B** and **C)** are hepatic and plasma ALT activities, respectively. (**D** and **E)** are hepatic and plasma AST activities, respectively. Data are presented as mean ± SEM, n = 6/group. The left side of each graph shows a single-factor analysis, while the right side shows a 2-factor analysis. On the left side, ^#^*P* < 0.05; ^##^*P* < 0.001 shows the difference from the control group according to the T-test. At the same time, different superscripts (a, b and c) indicate significant differences among the 4 HELP diet groups when there was significant interaction between CS and CC. According to 2 factors analysis from CS and CC, an asterisk (*) shows differences between the groups. CS**−** and CS+ without and with (100 mg/kg) cysteamine supplementation. CC**−** (choline 1182 mg/kg) and CC+ (choline 4,124 mg/kg) without and with supplementation of choline chloride, respectively. CS**−**CC**−** was a typical high-energy low-protein diet model.

The liver showed a typical color with no hemorrhages in control, CS+CC**−**, and CS**−**CC+ groups (Figure 5-Liver gross appearance). Results showed that with the addition of CS and CC or both (CS+CC+), the characteristic FLHS symptoms were alleviated. However, the lower side of the liver was affected in the CS+CC+ group, but it was much better in appearance than the CS**−**CC**−** (HELP) group. In addition, H&E staining showed that the adverse effects of the HELP diet on the hepatocytes were alleviated with CS and CC supplementation as only a small number of inflammatory lymphocytes (CS**−**CC+ group), with slight edema and loose cytoplasm (CS+CC**−** group) appeared in these groups (Figure 5-H&E staining). The overall pathological changes based on the 4-point scoring system showed that the liver of the CS**−**CC**−** (HELP) group was significantly (*P* < 0.05) damaged than the Cont group (Figure 5A). The analysis of the main factors showed that CC supplementation significantly (*P* < 0.05) reduced the overall pathological damage in the liver.

The experiment analyzed plasma and hepatic ALT and AST levels to analyze the degree of liver impairment under FLHS caused by the HELP diet. The plasma ALT level was significantly (*P* < 0.05) reduced in the CS**−**CC+ and CS+CC+ groups, while the hepatic ALT level was significantly (*P* < 0.05) reduced in the CS**+**CC+ group than the Cont group (Figures 5B and 5C).

From 2 facts analysis, the main factors showed that plasma ALT and hepatic ALT, AST levels were significantly (*P* < 0.05) decreased with CS and CC supplementation (Figure 5 B-D). In contrast, plasma AST level was significantly (*P* < 0.05) reduced with supplementation of CS only (Figure 5E). In addition, a significant (*P* < 0.05) interactive effect of CS×CC showed that plasma and hepatic AST and ALT levels were reduced in the CS+CC**−**, CS**−**CC+, and CS**+**CC+ groups as compared with the CS**−**CC**−** (HELP) group.

### Antioxidant Capacity

The analysis of the main factors exhibited that supplementation of CS significantly (*P* < 0.05) increased the plasma GSH-Px activity and tended (*P* = 0.087) to decrease the plasma MDA content. While supplementation of CC significantly (*P* < 0.05) increased and tended (*P* = 0.051) to increase the plasma SOD and plasma GSH-Px activities, respectively. Additionally, a significant (*P* < 0.05) interactive effect of CS×CC showed that plasma GSH-Px activity was increased in the CS+CC−, CS−CC+, and CS+CC+ groups as compared with the CS−CC− (HELP) group (Table 1).

The analysis of the main factors exhibited that supplementation of CS significantly (*P* < 0.05) increased hepatic GSH-Px and CAT activities. Meanwhile, supplementation of CC significantly (*P* < 0.05) increased hepatic CAT activities only. In addition, the significant (*P* < 0.05) interactive effect of CS×CC showed that hepatic CAT and GSH-Px activities were enhanced in the CS+CC−, CS−CC+, and CS+CC+ groups as compared with the CS−CC− (HELP) group, while the hepatic MDA contents were reduced in the CS+CC− and CS−CC+ groups as compared with the CS−CC− (HELP) group (Table 1).

### Production Performance

*First phase (1*–*6 wk)* There were no significant differences between the control and CS**−**CC**−** (HELP) groups in laying indexes (*P* > 0.05) during wk 1 to 6. However, the HELP diet numerically decreased the HHEP and HHE number by 11.03%, egg mass (kg/bird) by 12%, compared with the Cont group (Table 2). The analysis of the main factors showed that CS and CC supplementation numerically (*P* > 0.05) improve the laying performance of hens. A significant (*P* < 0.05) interactive effect of CS×CC showed increased HHEP (%), number of hen-housed eggs, and egg mass (kg/hen) in the CS+CC− group as compared with CS−CC− (HELP) group (Table 2). In addition, the interactive effect of CS×CC showed a tendency to increase HDEP (%) (*P* = 0.095) and egg mass (g/d) (*P* = 0.079) in the CS+CC−, CS−CC+, and CS−CC− groups as compared with the CS−CC− (HELP) group.

**Table 2:** Effect of coated cysteamine with or without choline chloride on production performance (1–6 wk) in laying hens under FLHS induced by HELP diet.

Treatments		HHEP, %	HDEP, %	No of HHE	Egg mass, kg/hen	Egg mass, g/d	ADFI, g/d	Feed:Egg	Unqualified egg rate, %
Control		73.2	73.2	30.7	1.653	39.4	105.0	2.82	0.80
CC**−**	CS**−** (HELP)	65.1^b^	68.6	27.3^b^	1.455^b^	36.5	99.9	2.80	3.21
	CS+	79.6^a^	79.6	33.4^a^	1.727^a^	41.1	96.6*	2.35	1.32
CC+	CS**−**	75.7^ab^	75.7	31.8^ab^	1.678^ab^	40.0	101.8	2.59	0.19
	CS+	71.7^ab^	71.7	30.1^ab^	1.575^ab^	37.5	100.0	2.74	0.69
SEM		2.40	1.87	1.01	0.048	0.84	1.47	0.09	0.53
Means of main effects	CS**−**	70.4	72.1	29.6	1.566	38.2	100.85	2.69	1.70
	CS+	75.7	75.7	31.8	1.651	39.3	98.29	2.54	1.01
	CC**−**	72.4	74.1	30.4	1.591	38.8	98.23	2.57	2.27
	CC+	73.7	73.7	31.0	1.626	38.7	100.91	2.66	0.44
*P-value*									
Main factors	CS	0.222	0.417	0.221	0.301	0.572	0.350	0.408	0.564
	CC	0.756	0.924	0.755	0.660	0.974	0.329	0.621	0.139
	CS×CC	0.039	0.095	0.039	0.029	0.079	0.800	0.105	0.327

HHEP, hen-housed egg production; HDEP, hen-day egg production; HHE, hen-housed eggs; ADFI, average daily feed intake.

Each group had 6 replicates (n = 6/group). *P < 0.05 shows the difference from the control group based on T-test. Whereas a, b, and c were used to show the difference among the 4 HELP groups when there was significant interaction CS×CC. CS**−** and CS+ without and with (100 mg/kg) cysteamine supplementation. CC**−** (choline 1,182 mg/kg) and CC+ (choline 4,124 mg/kg) without and with supplementation of choline chloride, respectively.

*Second phase (7*–*13 wk)* The CS**−**CC**−** (HELP) group decreased (*P* < 0.05) feed:egg ratio compared with the Cont group, but had no difference on other laying indexes (*P* > 0.05) during wk 7 to 13 (Table 3). The analysis of the main factors showed that CS and CC supplementation numerically (*P* > 0.05) improve the laying performance of hens. A significant (*P* < 0.05) interactive effect of CS×CC showed increased egg mass (g/d) in the CS+CC+ than in the CS+CC− group. In addition, the interactive effect of CS×CC showed a tendency (*P* = 0.052) to increase HHEP (%) and HDEP (%) in the CS+CC+ group as compared with the CS−CC− (HELP) group (Table 3).

**Table 3:** Effect of coated cysteamine with or without choline chloride on production performance (7–13 wk) in laying hens under FLHS induced by HELP diet.

Treatments		HHEP, %	HDEP, %	No of HHE	Egg mass, kg/hen	Egg mass, g/d	ADFI, g/d	Feed:Egg	Unqualified egg rate, %
Control		90.3	90.3	44.2	2.611	53.3	148.2	2.40	0.59
CC -	CS- (HELP)	89.1	89.8	41.3	2.350	51.0^ab^	126.0	2.12*	3.05
	CS+	87.0	87.0	42.6	2.361	48.2^b^	130.0	2.32	0.27
CC +	CS-	87.2	87.2	42.7	2.389	48.8^ab^	131.2	2.33	0.15
	CS+	93.4	93.4	45.8	2.580	52.7^a^	129.8*	2.12*	0.37
SEM		1.18	1.18	0.76	0.057	1.01	3.88	0.06	0.55
Means of main effects	CS-	88.5	88.5	42.0	2.370	49.9	128.6	2.22	1.60
	CS+	90.2	90.2	44.2	2.471	50.4	129.9	2.22	0.32
	CC-	88.4	88.4	42.0	2.356	49.6	128.0	2.22	1.662
	CC+	90.3	90.3	44.2	2.484	50.7	130.5	2.22	0.26
*P*-value									
Main factors	CS	0.448	0.448	0.128	0.295	0.731	0.856	0.995	0.314
	CC	0.390	0.390	0.111	0.186	0.488	0.725	0.974	0.272
	CS×CC	0.052	0.052	0.524	0.351	0.045	0.705	0.110	0.242

HHEP: hen-housed egg production; HDEP: hen-day egg production; HHE: hen-housed eggs; ADFI: average daily feed intake.

Each group had 6 replicates (n = 6/group). *P < 0.05 shows the difference from the control group based on T-test. Whereas a, b, and c were used to show the difference among the 4 HELP groups when there was significant interaction CS×CC. CS**−** and CS+ without and with (100 mg/kg) cysteamine supplementation. CC**−** (choline 1,182 mg/kg) and CC+ (choline 4,124 mg/kg) without and with supplementation of choline chloride, respectively.

*Overall Experimental period (1*–*13 wk)* There were no significant differences between the control and CS**−**CC**−** (HELP) groups in laying indexes (*P* > 0.05) during the whole period. However, no mortality was observed in the Cont group, while the survival rate was decreased (*P* > 0.05) to 94.44% in the CS**−**CC**−** (HELP) group. On overall bases, HHEP, HDEP, HHE number, egg mass kg/hen, and egg mass g/d were numerically (*P* > 0.05) decreased by 8.38, 2.97, 8.37, 10.76, and 5.59%, respectively, in the CS**−**CC**−** (HELP) group than the Cont group (Table 4). The analysis of the main factors showed that CS supplementation showed a tendency (*P* = 0.069) to improve the HHEP (6.14 %) and the number of HHE (6.13 %). However, all other parameters were numerically improved with CS or CC supplementation. In addition, the non-significant (*P* > 0.05) interactive effect of CS×CC showed improved production performance in the CS+CC−, CS−CC+, and CS+CC+ groups as compared with the CS−CC− (HELP) group (Table 4).

**Table 4:** Effect of coated cysteamine with or without choline chloride on production performance (1–13 wk) in laying hens under FLHS induced by HELP diet.

Treatments		HHEP, %	HDEP, %	No of HHE	Egg mass, kg/hen	Egg mass, g/d	ADFI, g/d	Feed:Egg	Unqualified egg rate, %	Survival rate, %
Control		82.4	82.4	74.9	4.264	46.9	116.8	2.54	0.64	100.0
CC -	CS- (HELP)	75.5	79.9	68.7	3.805	44.2	104.2	2.36	3.12	94.4
	CS+	83.6	83.6	76.1	4.088	44.9	104.6	2.33	0.73	100.0
CC +	CS-	81.9	81.9	74.5	4.067	44.7	107.6	2.43	0.16	100.0
	CS+	83.4	83.4	75.9	4.154	45.7	106.1	2.33	0.45	100.0
SEM		1.50	0.66	1.37	0.076	0.46	2.32	0.04	0.53	1.11
Means of main effects	CS-	78.7	80.9	71.6	3.936	44.5	105.9	2.39	1.64	97.2
	CS+	83.5	83.5	76.0	4.121	45.3	105.3	2.33	0.59	100.0
	CC-	79.5	81.8	72.4	3.947	44.6	104.4	2.35	1.92	97.2
	CC+	82.6	82.6	75.2	4.111	45.1	106.8	2.38	0.30	100.0
*P*-value										
Main factors	CS	0.069	0.276	0.069	0.139	0.465	0.894	0.564	0.391	0.329
	CC	0.229	0.707	0.229	0.188	0.600	0.576	0.797	0.189	0.329
	CS×CC	0.203	0.650	0.204	0.425	0.907	0.827	0.786	0.273	0.329

Each group had 6 replicates (n = 6/group). CS**−** and CS+ without and with (100 mg/kg) cysteamine supplementation. CC**−** (choline 1,182 mg/kg) and CC+ (choline 4,124 mg/kg) without and with supplementation of choline chloride, respectively. HHEP: hen-housed egg production; HDEP: hen-day egg production; HHE: hen-housed eggs; ADFI: average daily feed intake.

### Egg Quality Analysis

*First month (d 30)* Egg quality analysis showed a non-significant difference between the Cont and CS−CC− (HELP) groups; however, the yolk color index was higher (*P* < 0.05) in the CS−CC− (HELP) than in the Cont groups. The results indicate that the yolk color index, eggshell strength, and eggshell thickness were notably higher (*P* < 0.05) in the CS+CC− group and the yolk color index in the CS+CC+ group exceeded that of the Cont group (Table 5). The analysis of the main factors showed that CS supplementation had a tendency (*P* = 0.054) to improve the albumen height and significantly (*P* < 0.05) enhanced the haugh unit index. In contrast, CC supplementation showed a tendency to reduce the haugh unit index (*P* = 0.076) and eggshell strength (*P* = 0.073). The interactive effect (*P* < 0.05) of CS×CC showed that the CS−CC+ group decreased the albumen height than the CS−CC− and CS+CC+ groups and haugh unit index than all other groups (Table 5).

**Table 5:** Effect of coated cysteamine with or without choline chloride on egg quality (d 30) in laying hens under FLHS induced by HELP diet.

Treatments		Yolk wt, g	Yolk:Egg	Yolk color	Albumen height, mm	HU	Eggshell St, Kgf	Eggshell thickness, mm
Control		12.10	22.42	6.17	9.58	98.75	5.16	0.384
CC -	CS- (HELP)	12.41	22.68	6.83*	9.85^a^	99.70^a^	4.89	0.370
	CS+	12.26	23.06	7.33*	9.42^ab^	98.22^a^	6.04*	0.411*
CC +	CS-	12.40	23.02	7.17	7.00*^b^	83.18*^b^	4.90	0.382
	CS+	12.44	22.90	7.17*	10.08^a^	100.67^a^	4.66	0.374
SEM		0.06	0.12	0.21	0.56	3.25	0.24	0.007
Means of main effects	CS-	12.41	22.85	7.00	8.43	91.44^x^	4.90	0.376
	CS+	12.35	22.98	7.25	9.75	99.44^y^	5.35	0.392
	CC-	12.34	22.87	7.08	9.63	98.96	5.47	0.390
	CC+	12.42	22.96	7.17	8.54	91.93	4.78	0.378
*P*-value								
Main factors	CS	0.906	0.852	0.435	0.054	0.046	0.227	0.137
	CC	0.857	0.894	0.793	0.107	0.076	0.073	0.291
	CS×CC	0.840	0.719	0.435	0.013	0.020	0.070	0.058

Each group had 6 replicates (n = 6/group). *P < 0.05 shows the difference from the control group based on T-test. “x and y” show the difference within factor CS. Whereas a, b, and c were used to show the difference among the 4 HELP groups when there was significant interaction CS×CC. CS**−** and CS+ without and with (100 mg/kg) cysteamine supplementation. CC**−** (choline 1182 mg/kg) and CC+ (choline 4124 mg/kg) without and with supplementation of choline chloride, respectively. HU: haugh unit.

*Second month (d 60)* At d 60 of the experiment, no significant (*P* > 0.05) effect of treatments was observed on all parameters of egg quality except yolk weight, which was lower in the CS+CC**−** than in the Cont group (Table 6). According to the main factors analysis, CC supplementation numerically increased the yolk:egg ratio, yolk color index and eggshell strength. No interactive effect (*P* > 0.05) of CS×CC was observed.

**Table 6:** Effect of coated cysteamine with or without choline chloride on egg quality (d 60) in laying hens under FLHS induced by HELP diet.

Treatments		Yolk wt, g	Yolk:Egg	Yolk color	Albumen height, mm	HU	Eggshell St, Kgf	Eggshell thickness, mm
Control		14.55	25.24	6.50	9.00	94.93	4.72	0.377
CC -	CS- (HELP)	14.22	24.10	6.17	10.23	100.43	4.71	0.387
	CS+	13.45*	24.11	6.50	9.13	96.17	4.84	0.397
CC +	CS-	13.85	24.83	6.83	9.62	98.42	5.27	0.395
	CS+	13.73	24.76	6.17	9.50	97.78	4.43	0.371
SEM		0.19	0.22	0.13	0.22	0.95	0.14	0.005
Means of main effects	CS-	14.04	24.47	6.50	9.93	99.43	4.99	0.391
	CS+	13.59	24.43	6.33	9.32	96.98	4.63	0.384
	CC-	13.84	24.11	6.33	9.68	98.30	4.78	0.392
	CC+	13.79	24.80	6.50	9.56	98.10	4.85	0.383
*P*-value								
Main factors	CS	0.229	0.954	0.604	0.209	0.266	0.512	0.667
	CC	0.903	0.275	0.604	0.792	0.927	0.896	0.667
	CS×CC	0.378	0.943	0.130	0.306	0.407	0.379	0.392

Each group had 6 replicates (n = 6/group). *P < 0.05 shows the difference from the control group based on T-test. CS**−** and CS+ without and with (100 mg/kg) cysteamine supplementation. CC**−** (choline 1182 mg/kg) and CC+ (choline 4124 mg/kg) without and with supplementation of choline chloride, respectively. HU: haugh unit.

*Third month (d 90)* Egg quality analysis exhibited a non-significant (*P* > 0.05) difference between the Cont and CS−CC− (HELP) groups; however, the yolk color index was higher (*P* < 0.05) in the CS−CC+ and CS+CC+ groups than the Cont group (Table 7). The analysis of the main factors exhibited that CS addition significantly (*P* < 0.05) enhanced the eggshell strength. Meanwhile, CC supplementation showed a tendency (*P* = 0.083) to increase the yolk weight and significantly (*P* < 0.05) increase the yolk:egg ratio and yolk color index. Similarly, the interactive effect of CS×CC showed a tendency (*P* = 0.098) to enhance the albumen height in the CS+CC+ than in the CS−CC− (HELP) group (Table 7).

**Table 7:** Effect of coated cysteamine with or without choline chloride on egg quality (d 90) in laying hens under FLHS induced by HELP diet.

Treatments		Yolk wt, g	Yolk:Egg	Yolk color	Albumen height, mm	HU	Eggshell St, Kgf	Eggshell thickness, mm
Control		15.33	25.72	6.33	9.98	99.17	4.06	0.359
CC -	CS- (HELP)	14.88	25.08	6.33	10.12	99.03	3.61	0.350
	CS+	14.50	25.60	6.50	9.25	96.35	5.10	0.382
CC +	CS-	14.97	26.61	7.17*	9.43	97.68	3.91	0.360
	CS+	15.73	27.00	7.50*	11.28	104.02	4.98	0.354
SEM		0.21	0.35	0.24	0.36	1.30	0.30	0.005
Means of main effects	CS-	14.92	25.85	6.75	9.78	98.36	3.76^x^	0.355
	CS+	15.12	26.30	7.00	10.27	100.18	5.04^y^	0.368
	CC-	14.69	25.34^n^	6.42^n^	9.68	97.69	4.35	0.366
	CC+	15.35	26.81^m^	7.33^m^	10.36	100.85	4.45	0.357
*P*-value								
Two factors	CS	0.598	0.366	0.376	0.537	0.605	0.020	0.472
	CC	0.083	0.007	0.003	0.399	0.374	0.857	0.533
	CS×CC	0.131	0.898	0.766	0.098	0.209	0.681	0.275

Each group had 6 replicates (n = 6/group). *P < 0.05 shows the difference from the control group based on T-test. “x and y” and “n and m” show the difference within factor CS and CC, respectively. CS**−** and CS+ without and with (100 mg/kg) cysteamine supplementation. CC**−** (choline 1182 mg/kg) and CC+ (choline 4124 mg/kg) without and with supplementation of choline chloride, respectively. HU: haugh unit.

## DISCUSSION

Fatty liver hemorrhage syndrome is a metabolic disorder of laying hens caused by factors such as nutrition, genetics, and environment. Literature suggests that FLHS occurs in up to 16% of laying hens globally, impacting their production and causing potential deaths, resulting in significant economic losses for the industry (Rozenboim et al., 2016). The HELP diet has been identified as a potential contributor to FLHS (Rozenboim et al., 2016; Gao et al., 2019; Miao et al., 2021). The HELP diet is a natural inducer of FLHS as it promotes adipogenesis by providing higher energy intake while impeding lipoprotein synthesis due to the limited availability of dietary protein. This ultimately hinders the fat removal process from the liver, resulting in triglyceride accumulation in the liver. The current study had set an FLHS model using HELP in laying hens and tried to explore the impacts of CS and CC on alleviating FLHS and reveal the mechanism behind them.

The present experiment showed that abdominal fat and liver index percentages were increased under the FLHS model than the Cont, and our results are consistent with previous findings (Miao et al., 2021). The release of high levels of unesterified fatty acids from adipose tissues can result in fat accumulation in the abdominal region and liver. Disruption of the liver's equilibrium concerning fat formation and metabolism can cause hepatocyte damage, accumulating fat in the liver and ultimately resulting in FLHS. CS influences the enzymes involved in lipid metabolism directly and has a mitigating effect on fat accumulation (Tao et al., 2020). By increasing the activities of hormone-sensitive lipase and regulating the activities of fatty acid synthase, CS could potentially reduce abdominal fat deposition in the context of the HELP diet. Previous studies have suggested that choline could significantly reduce fat content in the liver of laying hens (Dong et al., 2019) and broiler breeders (Rama Rao et al., 2001). The present research showed that simultaneous supplementation of both CS and CC to the HELP model could decrease the abdominal fat percentage to the level of the Cont group. The liver index percentage at simultaneous supplementation of both CS and CC was higher than the Cont group, but its TG and T-CHO were not increased. A high liver index in poultry without excessive fat accumulation can arise from various factors, indicating nutritional imbalances, potential health issues or stressors affecting liver function (Anene et al., 2023). As in this experiment, the HELP diet produced stress on hens, as shown in the oxidative stress section, but CS and CC tried to avoid this stress and support the hens to survive in stressful conditions. Therefore, the HELP diet applied in the experiment successfully established the FLHS model, and the supplementation of CS and CC was found to significantly reduce the pathological changes associated with FLHS induced by the HELP diet.

Higher dietary energy levels could enhance the plasma fatty acids contents, which elevates the lipogenesis in the liver and becomes the cause of TG accumulation in the hepatocytes, directing to FLHS (Whitehead, 1979). In the current study, the plasma and hepatic TG levels and Oil Red O staining analysis of the liver all indicated lipid accumulation with the HELP diet in the CS−CC− (HELP) group. As predicted, the supplementation of CS and CC relieved lipid accumulation in the liver by decreasing hepatic TG levels, which was consistent with the Oil Red O staining of liver tissues. This may be due to the fact that CS has a direct effect on enzymes involved in lipid metabolism and has a reducing effect on fat deposition. CS could increase the activity of hormone-sensitive lipase, which is involved in the hydrolysis of stored TG, and decrease the activity of fatty acid synthase, which participates in fat synthesis in adipose tissue (Tao et al., 2020). In addition, choline is the component of phosphatidylcholine that is involved in the synthesis of VLDL (Aziza et al., 2019) required to transfer fat from the liver to the blood (Vance, 2008), which could be the reason for reduced fat deposition with CC supplementation in the current study. Taken together, the supplementation of CS and/or CC is helpful for alleviating lipid accumulation in liver of laying hens under FLHS model.

Lipoprotein is the carrier of lipids between the liver and peripheral blood. Elevated LDL-C level is a causative agent of cardiovascular disease and a risk factor linked with atherosclerosis (Ridker, 2014). In contrast, HDL-C is the “favorable cholesterol” that scavengers to remove unnecessary cholesterol from the bloodstream (Jacobs et al., 2017; Khera et al., 2017). The balance of HDL-C and LDL-C is required to balance lipid metabolism (Zhang et al., 2019), and a disorder in this equilibrium could cause a fatty liver to occur. In the present study, the T-CHO and LDL-C were increased, but the HDL-C was reduced in the CS−CC− (HELP) group, and supplementation of CS and CC reversed these indices. Collectively, it could be stated that the HELP diet increased the deposition of TG and disturbed the HDL-C and LDL-C balance, and CS and CC supplementation helped the hens resist these changes. One of the key contributors to the progression of FLHS is insulin resistance (Zhuang et al., 2019), while adiponectin is an insulin-sensitizing and anti-inflammatory agent (Peake et al., 2005; Shetty et al., 2009) which plays a vital role in maintaining glucose and lipid homeostasis (Yamauchi et al., 2002). In addition, adiponectin has also been reported to enhance the phosphorylation of acetyl-CoA carboxylase and fatty-acid oxidation in isolated hepatocytes (Yamauchi et al., 2002). In the current experiment, the HELP diet model decreased plasma adiponectin contents, which was consistent with its higher lipid accumulation in the liver. Down-regulation of adiponectin in the HELP diet model might be due to oxidative stress (Arita et al., 1999; Kamada et al., 2008). Meanwhile, the CS and CC supplementation alone successfully recovered it to the level of the control group, and the impact of CS was more robust than CC. This result indicated that supplementing with CS and CC alone has the potential to improve insulin sensitivity by increasing adiponectin levels, thereby helping to maintain LDL-C and HDL-C levels and preventing the buildup of T-CHO and TG.

Using HELP diets for extended periods produces immoderate reactive oxygen species (ROS) (Lee et al., 2019). The antioxidants (GSH-Px and T-SOD) oppose excessive ROS production, but unrestrained production of ROS under limited availability of antioxidants upsets the oxidative equilibrium, resulting in oxidative stress that further worsens the FLHS (Zhang et al., 2008; Lee et al., 2019). MDA is the metabolite of lipid peroxidation, and its elevated level appears as a sign of oxidative instability. According to the previous studies, the results of the present study showed that FLHS significantly reduced the anti-oxidative status of laying hens, as depicted by elevated MDA and reduced SOD and GSH-Px concentrations. In the current study, the supplementation of CS improved antioxidant capacity by increasing GSH-Px in plasma and CAT and GSH-Px in liver, while the supplementation of CC improved antioxidant capacity by increasing SOD in plasma and CAT in liver under the FLHS model. Those results were consistent with the previous studies that done with CS and CC in animals. Literature suggests that CS protects cells from oxidative stress (Vasin, 2014) and can neutralize oxidative stress in animals (Barnett and Hegarty, 2016). The biosynthesis of CS up-regulated during oxidative stress suggested that CS protects the cells from this stress (Kobayashi et al., 2006). CS also increases the intracellular concentration of GSH-Px as CS can cross cell membranes (Prescott and Critchley, 1983). In addition, CS is one of the few bio-molecules that can directly remove toxic metabolites of lipid peroxidation known as ROS (Winterbourn and Hampton, 2008). Additionally, choline, through its metabolite betaine, could improve methionine biosynthesis, enhancing the production of GSH-Px and hepatic redox status in laying hens (Zeisel and Blusztajn, 1994). Literature evidence that oxidative stress causes insulin resistance, which leads to different metabolic disorders (Maddux et al., 2001; Henriksen et al., 2011). So, the adding of CS and CC had a potential to decreases the insulin resistance by relieving oxidative stress, which could be attributed to the increase of plasma adiponectin levels of hens under FLHS disease in the CS (CS+CC-) and CC (CS-CC+) supplementation groups. Hence, it could be suggested that CS and CC supplementation could ameliorate the oxidative stress of laying hens under FLHS disease.

In this experiment, the HELP diet turns the liver yellowish-brown and greasy with hemorrhages and bleeding spots on its surface, which are natural symptoms of FLHS (Mete et al., 2013). In addition, plasma and hepatic levels of ALT and AST were examined to determine the extent of liver injury caused by the HELP diet-induced FLHS. These aminotransferases are significant indicators of hepatocyte damage. AST and ALT enzyme activities are also utilized to indicate FLHS in poultry, as per Rozenboim et al. (2016). The analysis revealed that the ALT and AST activities were significantly higher in the HELP group, showing intense hepatocyte injury in hens under FLHS, as previously reported by Zhang et al. (2019) and Gao et al. (2019). However, the dietary supplementation of CS and CC to HELP diets showed a significant reduction in the AST and ALT levels, indicating that CS and CC can protect the liver against FLHS induced by the HELP diet, which might be due to lower oxidative stress and decreased lipid accumulated in the liver.

Recent studies showed that FLHS is mainly accompanied by ovarian and oviduct degeneration, leading to a reduced rate of egg production (Rozenboim et al., 2016; Xing et al., 2020). During wk 1 to 6, the laying rate and egg mass (kg/bird) in the HELP group were reduced by 11.03% and 12% compared with the Cont group. Laying performance parameters were also affected during the overall experiment period. Previous studies also suggested reducing the laying rate under FLHS due to the HELP diet (Xing et al., 2020; Miao et al., 2021). Consistent The supplementation of CS (CS+CC−) rescued that phenomenon and showed higher HHEP, HDEP, No of HHE, and egg mass than those in the CC-CS- (HELP) group during wk 1 to 6. In the whole period (wk 1–13), the addition of CS also tended to increase HHEP, HDEP, and No of HHE. The results suggested that CS supplementation independently restores the production performance of laying hens during the early period, which contributed to better production performance in the whole period. Furthermore, the current study found that the best production performance during wk 7 to 13 was in the CC+CS+ group, indicating that the combination of CS and CC is more advantageous for hens in subclinical FLHS state for a long term. These results suggested that negative impacts on production performance caused by FLHS could be alleviated with dietary CS and CC supplementing in laying hens.

In addition, the supplementation of CS and CC based on the HELP diet also showed some changes in egg quality of laying hens. On d 30, the increasing CC without CS (CS−CC+) had the lowest albumen height and HU, but the addition of CS without CC (CS+CC−) had the highest eggshell strength and eggshell thickness. Results on d 90 showed that the increasing of CC increased yolk weight, yolk:egg, and yolk color, while the addition of CS increased eggshell strength. The above results suggested that the supplementation with CS had positive effects on egg quality, but the independently increasing of CC only benefit for the egg quality after a long term FLHS tolerance. The above changes may be attributed to CS and CC's ability to provide support against oxidative stress and prevent fat accumulation in the abdominal cavity and liver.

## CONCLUSIONS

The current investigation has validated the substantial influence of CS and CC supplementation in ameliorating FLHS in laying hens. The introduction of CS and increasing the CC in the diet enhance the anti-oxidative capacity and deter the fat accumulation. Consequently, early-stage (wk 1–6) supplementation with CS exhibits superior production performance and egg quality. In contrast, the augmentation of dietary CC levels alongside CS supplementation emerges as a more favorable approach for averting the deleterious effects of prolonged FLHS in laying hens.

## DISCLOSURES

The authors declare no conflicts of interest.

## Appendix Supplementary materials

Supplementary Table 1: Ingredients composition and nutrients profile of basal and high-energy low-protein diets. Supplementary Table 2: Details of the 4-point scoring system to evaluate the pathological changes in liver tissues. Image, application 1

## Footnotes

Supplementary material associated with this article can be found in the online version at doi:10.1016/j.psj.2024.104296.
