# Supplementary material for: Coated cysteamine and choline chloride could be potential feed additives to mitigate the harmful effects of fatty liver hemorrhagic syndrome in laying hens caused by high-energy low-protein diet
Source: Poult Sci. 2024 Sep 5;103(12):104296. doi: 10.1016/j.psj.2024.104296 (PMC11437759; doi:10.1016/j.psj.2024.104296)
Supplement: Supplementary file 1 — Supplementary Table 1: Ingredients composition and nutrients profile of basal and high-energy low-protein diets. Supplementary Table 2: Details of the 4-point scoring system to evaluate the pathological changes in liver tissues. [file mmc1.docx]

**Supplementary Table 1** Composition and nutrient levels of the diets for laying hens.

| Composition of diet  (air-dry basis) % | Basal diet | High-energy low-protein diet |
| --- | --- | --- |
| Corn | 64.00 | 70.00 |
| Wheat bran | 2.00 | 1.20 |
| Soybean meal | 24.00 | 14.58 |
| Fat-soybean oil | 0.00 | 4.22 |
| Calcium | 8.00 | 8.00 |
| Premix* | 2.00 | 2.00 |
| Total | 100.00 | 100.00 |
| Nutrient level |  |  |
| Crude Protein | 15.86 | 12.00 |
| Available Phosphorus | 0.51 | 0.46 |
| Arginine | 1.03 | 0.74 |
| Methionine | 0.37 | 0.32 |
| Valine | 0.77 | 0.58 |
| Metabolic energy (kcal/kg) | 2678.99 | 3100.00 |
| Met + Cys | 0.67 | 0.56 |

The basal diet was formulated according to the National Research Council (1994). FLHS was induced by the high-energy low-protein diet. The control and FLHS group received basal diet and the high-energy low-protein diet for 13 weeks, respectively. *The ingredient of premix: multiple vitamins, 30 mg; cupric sulfate, 4.6 mg; ferrous sulfate, 28.4 mg; manganous sulfate, 35.46 mg; zinc sulfate, 76 mg; zeolite powder, 6 mg; sodium selenite, 5 mg; anti-oxidizing quinolone, 50 mg; choline, 90 mg; bacitracin zinc, 26.7 mg; bran, 350 mg; methionine,100 mg.

**Supplementary Table 2** Illustration of the 4-point scoring system for evaluation of pathological changes in liver tissues.

| Numerical Score | Description | Definition |
| --- | --- | --- |
| 0 | Within normal limits | Tissue considered to be normal, under the conditions of the study and considering the age, sex, and strain of the animal concerned. Alterations may be present, which, under other circumstances, would be considered deviations from normal. |
| 1 | Minimal | The amount of change present barely exceeds that which is considered to be within normal limits. |
| 2 | Slight | In general, the lesion is easily identified but of limited severity. |
| 3 | Moderate | The lesion is prominent, but there is significant potential for increased severity. |
| 4 | Severe | The degree of change is as complete as possible (occupies the majority of the organ). |
